# Changes in health behaviors and conditions during COVID-19 pandemic strict campus lockdown among Chinese university students

**DOI:** 10.3389/fpsyg.2022.1022966

**Published:** 2022-10-17

**Authors:** Boyi Zhang, Si Man Lei, Shenglong Le, Qiang Gong, Sulin Cheng, Xiuqiang Wang

**Affiliations:** ^1^Department of Physical Education, Shanghai Jiao Tong University, Shanghai, China; ^2^Faculty of Education, University of Macau, Taipa, Macao SAR, China; ^3^Exercise Translational Medicine Center, National Center for Translational Medicine, Shanghai Jiao Tong University, Shanghai, China; ^4^Department of Physical Therapy, Taihe Hospital, Hubei University of Medicine, Shiyan, China; ^5^Faculty of Sport and Health Sciences, University of Jyväskylä, Jyväskylä, Finland; ^6^Key Laboratory of Systems Biomedicine (Ministry of Education), Shanghai Center for Systems Biomedicine, Shanghai Jiao Tong University, Shanghai, China

**Keywords:** COVID-19, university students, mental health, physical activity, sleep disorders, strict lockdown

## Abstract

**Objective:**

To explore how a stringent campus lockdown affects the physical activity (PA), sleep and mental health of Chinese university students living in student dormitories during the COVID-19 pandemic.

**Methods:**

Data on PA, sleep and mental health were collected between 24 March and 4 April 2022 from 2084 university students (mean age = 22.4 years, 61.1% male students) *via* an online questionnaire distributed by the students’ advisers of each dormitory. The Chinese short version of the International Physical Activity Questionnaire (IPAQ-C), Athens Insomnia Scale (CAIS) and General Health Questionnaire 12-item (GHQ-12) were applied. The Mann–Whitney test and Kruskal-Wallis tests were used to evaluate the PA profile differences between genders, before and during the lockdown period and between students’ living environments. Chi-squared (*χ*2) or Fisher’s exact test was used to assess changes in health behaviors by gender and students’ living environment compared to before the lockdown. A mediation model was used to examine whether sleep disorder mediated the relationship between PA and mental health in different students’ living environments.

**Results:**

Participants reported a significant decrease in weekly total PA levels (63.9%). Mean daily sedentary time increased by 21.4% and daily lying time increased by 10.7% compared to before lockdown. Among the participants, 21.2% had experienced insomnia, and 39.0% reported having high mental distress. Female students reported 10% higher rates of sleep disorders than male students (*p* < 0.001), and also experienced a higher incidence of mental disorders (*p* < 0.001). Students living with three roommates had a larger decrease in frequencies and durations of participation in light PA than other students (*p* < 0.001). PA was negatively associated with sleep and mental health, and sleep disorder was a mediating factor between PA and mental health in the students living with two and three roommates.

**Conclusion:**

This study showed that strict lockdowns within university dormitories during the COVID-19 pandemic had a negative effect on the health of university students by changing their health behaviors, physical activity and sleep. Our findings indicate a need for strategies to promote an active lifestyle for students in space-limited dormitories in order to maintain health during a prolonged lockdown.

## Introduction

The coronavirus disease (COVID-19) has brought dramatic political and economic changes around the world since the outbreaks in late 2019. The frequency and stringency of prevention measures vary from country to country as the outbreak continues to develop (Hale et al., 2020). Although more than 2 years have passed, conditions of strict confinement in place to control the pandemic themselves present a challenge to the physical and mental health of diverse populations (Odone et al., 2020; World Health Organization, 2022). On 26 November 2021, the WHO classified Omicron as a new variant of the SARS-CoV-2 (World Health Organization, 2021). Through observations and lessons learned from the strained healthcare systems in Hong Kong and Shenzhen, Shanghai adopted a strict and extensive containment control strategy to prevent the spread of Omicron following the emergence of Omicron cases (Zhang et al., 2022). Schools and universities in Shanghai complied with the policy of closing campuses (Shanghai Municipal Health Commission, 2022a) and students had to switch to learning online and dormitory quarantine with necessities delivered by volunteers. The only outdoor activities were walking to the nucleic acid PCR testing stations nearby.

According to the Shanghai Municipal Health Commission, as of 31 March 2022, 32,648 cases of asymptomatic infection, 1,937 cases have been identified, and 1,340 people have treated in hospital (Shanghai Municipal Health Commission, 2022b). A systematic review and meta-analysis showed that the mental health of university students can be influenced by factors such as national policy and the survey date (Li et al., 2021). Negative moods as a result of isolation and stressors create potential physical and psychological health risks among university students, as has been reported previously (Li et al., 2021). The stresses and restrictions associated with strict confinement due to the COVID-19 outbreak put university students at greater risk of adverse impacts on their physical activity (PA), academic achievement, social interactions, future careers and personal opportunities (Zhang et al., 2022). Based on the stress buffer hypothesis (Cohen and Wills, 1985), social support can protect individuals from the potentially pathogenic effects of stress. This hypothesis assumes increased physical activity can buffer the negative health effects of stress, and maintaining physical activity during a high-stress period may reduce its negative impact on students’ sleep and mental health (Hamer et al., 2006; Tsatsoulis and Fountoulakis, 2006; Gerber and Pühse, 2009). While Sudden outbreaks may make university students more prone to changes in PA (Wang et al., 2020) and psychological stress (Serafini et al., 2012; Zhang et al., 2020).

Numerous studies have found that physical activity and sleep were important factors that influence mental health, with increased physical activity having a direct or indirect positive effect on sleep and psychological well-being (Baglioni et al., 2011; Ghrouz et al., 2019; Trabelsi et al., 2021). In a randomized controlled trial, Scott et al. found that sleep disturbance may be at the root of mood disorders and depression (Scott et al., 2017). Low levels of physical activity during COVID-19 were found to be highly associated with an increased risk of mental disorders in a Brazilian study (Puccinelli et al., 2021). Gender differences were also found in facing the psychological stress caused by COVID-19. And recent empirical research regarding gender differences in mental and physical health found that females perceived greater anxiety and depressive symptoms among university students in Iceland (Gestsdottir et al., 2021). Likewise, the latest investigation indicated negative impacts of COVID-19 home isolation, and females reported a higher prevalence of anxiety (Bigalke et al., 2020).

Differences in living environments can intensify changes in people’s lifestyles during the period of confinement. In a typical university dormitory in China, 4–8 students share a 20^2^ m living space. Living in small apartments (<60^2^ m) and having inadequate privacy were strongly correlated with moderate-to-severe and severe depressive symptoms during a COVID-19 lockdown (Amerio et al., 2020). Furthermore, evidence suggests that sleep quality is critical in modulating the effects of PA on mental health (Wunsch et al., 2017; Wang et al., 2020), but the correlation between PA, sleep, and mental health during a period of lockdown remains controversial. Wilson et al. (2021) found that PA and mental health among US college students declined significantly during the COVID-19 pandemic compared to before the pandemic, but PA did not affect mental health. On the other hand, one study from South Africa found that PA affected mental health outcomes during the lockdown, and low PA predicted greater insomnia symptom severity, which in turn predicted increased depressive-and anxiety-related symptoms (Lewis et al., 2021). However, little is known about the effects of the number of students sharing a dormitory during a period of campus lockdown on PA, sleep, and mental health.

With increasingly stringent quarantine requirements, understanding the changes in PA, sleep and health behavior of university students before and after the COVID-19 outbreak lockdown may guide to subsequent development of isolation policy to minimize the adverse effects. Therefore, the purpose of this study is to, (1) describe the changes in university students’ PA, sleep, mental health and health behavior condition patterns in dormitories during and before lockdown; (2) assess the differences in these parameters across the different number of roommates in university students; (3) investigate the relationships among PA, sleep and mental health and how they relate to the different numbers of students sharing a dormitory. Based on previous studies, our main hypothesis is that the PA levels of university students under strict campus lockdown will be significantly reduced and that low PA levels will be highly correlated with worse sleep and mental health. Moreover, this relationship varies across university students’ living environments (number of roommates). It was anticipated that this study could provide useful PA, sleep, and psychological advice for university students and authorities in responding to such public health emergencies in the future.

## Materials and methods

### Study design and procedure

The study was a cross-sectional design with a convenience sample that targeted students studying and living on the (Minhang district) campus of Shanghai Jiao Tong University. The study was launched on March 24, 2022, which is 2 weeks after the start of the enforced quarantine on campus. We collected data on PA, sedentary time, insomnia symptoms, mental health and sociodemographic information using a commercial online survey platform (i.e., Wen Juan Wang).^1^ Electronic informed consent was obtained from each participant prior to the beginning of the survey. Eligible participants were contacted through the students’ advisers of each dormitory, and about 10% of students from each dormitory (a total of 69 dormitories) was involved. A QR code of the questionnaire was provided *via* WeChat. The questionnaire was anonymous and produced de-identified data. The participants were volunteers without a monetary incentive, and they were informed about the use of their information. After the questionnaires were obtained, a researcher reviewed the collected questionnaire to ensure the quality of the responses. The structured questionnaire included four sections: (1) socio-demographic characteristics (i.e., age, height, weight, gender, college degree, discipline and number of roommates), (2) health behavior and condition changes (i.e., screen time, body weight change, sleep behavior, etc.,) compared to 2 weeks before the lockdown, and (3) the condition of PA, SB before and during the lockdown, (4) sleep disorder and mental health during the lockdown. The study was performed according to the Declaration of Helsinki and was approved by the ethics committee of Shanghai Jiao Tong University, China (B2022185M).

### Participants

The participants were from the Shanghai Jiao Tong University who were currently stated at the university. The inclusion criteria were that participants were aged older than 18 years, could read and understand the Chinese language and the purpose of the survey. We exclude questionnaires with apparent errors (e.g., sum of all activities hours exceeding 24 a day) and participants who subjectively reported physical or mental disability.

### PA and SB

The Chinese short version of the International Physical Activity Questionnaire (IPAQ-C) was used, and has good reliability across domains (ICC: from 0.81 to 0.89) and moderate validity (Deng et al., 2008). The Chinese version consists of 7 items recall measure of 7 days. The items were modified relative to before and during campus lockdown (i.e., “what is your vigorous PA level during the campus lockdown?”) For the level of PA, participants reported the frequency (days per week) and duration (hours and minutes) of vigorous, moderate and light PA performed previously and during the lockdown weeks (Wang et al., 2020). The calculation of continuous scores followed the IPAQ–SF scoring protocol (Committee, 2005) to estimate the weekly Metabolic Equivalent of Task (MET) by multiplying the minutes of days, days per week, and MET value of light, moderate and vigorous levels of PA (3.3, 4.0 and 8.0 METs, respectively). Then the participants’ PA was categorized into three levels: light (<600 MET minutes/week), moderate (600–1,499 MET minutes/week), and vigorous(≥1,500 MET minutes/week) based on the total metabolic equivalents (METs) per week (Lee et al., 2011). In addition, the participants were asked to report SB time by stating the time in hours and minutes spent in sitting and lying down during and before the lockdown (i.e., “How many hours do you sit in a 24-h day?,” “How many hours do you lying down in a 24-h day?”).

### Athens insomnia scale

The Chinese version of the Athens Insomnia Scale (CAIS) was used to assess sleep disorders and particularly insomnia symptoms and consists of eight self-administered items (i.e., *“*During the recent past, how was your total sleep duration?,” “How was your sense of well-being during the day?”) (Chiang et al., 2009). The item scoring of the CAIS uses a 4-point Likert scale, rating from 0 to 3, and the total scores are calculated values from 0 to 24. According to the original AIS version (Soldatos et al., 2003), the optimal cutoff is set at 6 or higher to discriminate and identify a person with severe insomnia symptoms. Higher scores suggest severe insomnia symptoms, with lower scores indicating fewer insomnia symptoms. The CAIS has demonstrated good validity and reliability with the Cronbach’s a of internal consistency of 0.84 and a test-rest reliability of 0.86 (Chiang et al., 2009), and Cronbach’s alpha coefficient for CAIS was 0.82 in the present sample.

### General health questionnaire

Mental health was assessed using the Chinese version of the General Health Questionnaire 12-item (GHQ-12; Chan and Chan, 1983). Items 2, 5, 6, 9, 10, and 11 are negatively phrased. The original and preferable Goldberg bimodal scoring method (0-0-1-1) was applied, and higher total scores (0–12) represented poorer mental health/psychological distress (Goldberg and Hillier, 1979; Goldberg and Williams, 1988). The items concern a variety of psychological constructs, such as anxiety, depression, and social dysfunction (i.e., “Have you recently been feeling unhappy and depressed?,” “Have you recently been losing confidence in yourself?”). A factor score is calculated by adding the item score, and values span 0–12, with higher scores indicating poorer mental health. The GHQ-12 has demonstrated both convergent validity (*r* = 0.47–0.76, *p* < 0.05) and internal consistency (Cronbach’s *α* = 0.89) (Zhong et al., 2021). Cronbach’s alpha coefficient for GHQ-12 was 0.85 in the present sample.

### Statistical analyzes

The Shapiro–Wilk test was used to check the normality of data distribution. If data were not normally distributed, they were analyzed with non-parametric analysis methods. Descriptive statistics were used to present the continuous data as the mean and 95% confidence interval (CI) and categorical data as numbers (*n*) and proportions (%). The Mann–Whitney test was used to evaluate the PA profile differences between genders, and between before and during the lockdown period. Kruskal-Wallis tests were used to compare the different PA variables between students’ living environments. We stratified the analysis between males and females and between students’ living environment profiles. A cross-table Chi-squared (*χ*^2^) or Fisher’s exact test were used where appropriate to assess the changes in health behaviors by gender and students´ living environment compared with before the lockdown, and Cramer’s V for estimation of effect size (small, medium or large) (McHugh, 2013; Kim, 2017). Change rates (△%) in PA profiles were calculated by subtracting the value during the lockdown from the value before the lockdown, and then dividing it by the value before the lockdown.

**Figure 1:**
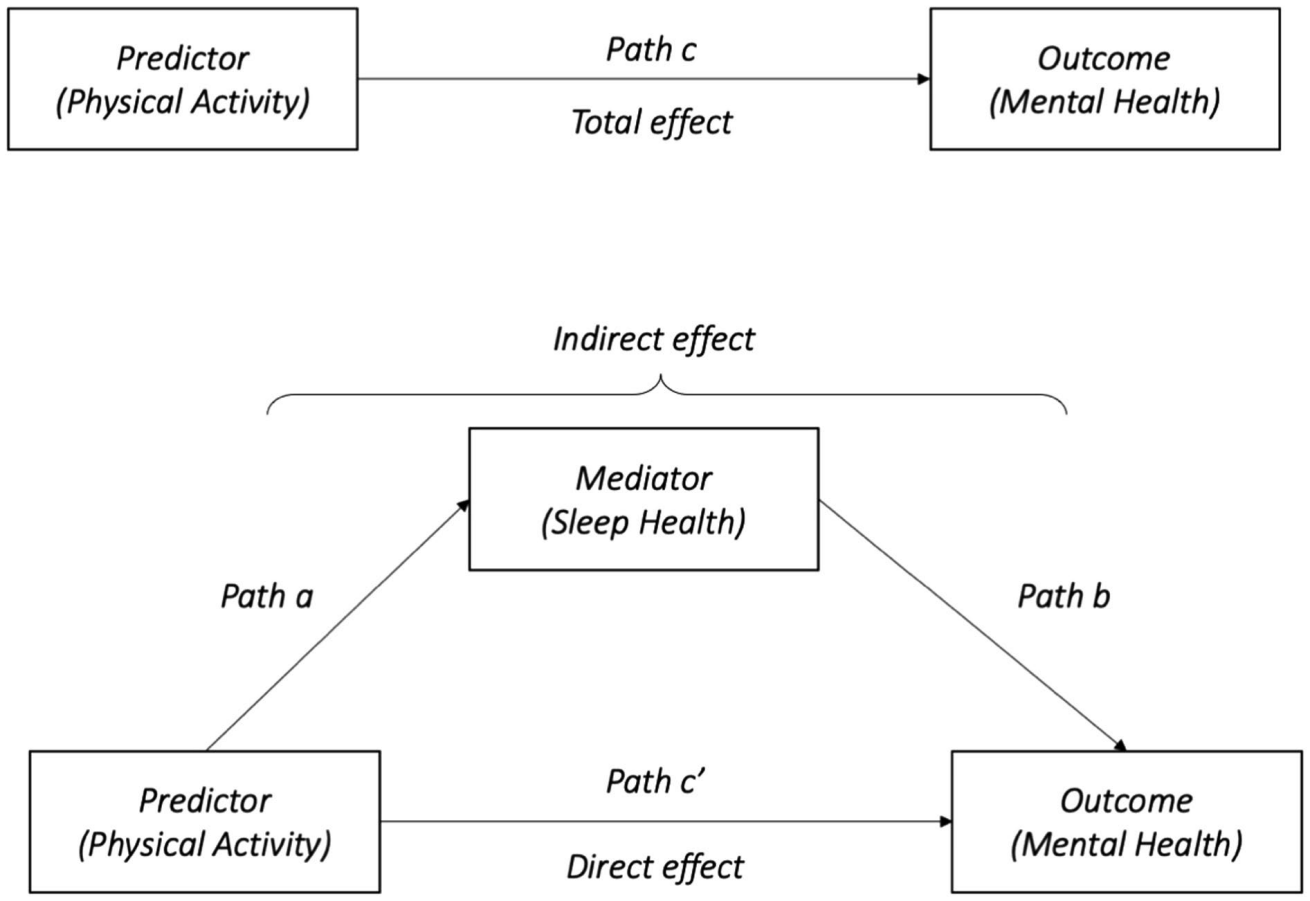
Conceptual model: how sleep disorder mediates the association between physical activity and mental health. a, b, c, and c’ refer to the path of models.

In addition, mediation models were used to test whether sleep disorder mediated the relationship between PA and mental health with different numbers of roommates, adjusted for age and gender. According to Zhao et al. (2010), we chose the indirect path a*b test as the first step to estimate the mediating effect. In the mediation models, PA was considered as an independent variable, while sleep disorder was used as the mediator variable and mental health was treated as a dependent variable (Figure 1). Three regression models were established to verify the mediating effect. Coefficients for the regression of PA on mental health were calculated in the first regression model (path a). The regression coefficients of PA and sleep disorder were calculated in the second regression model (paths b and c’). The predictor effect of PA on mental health excluding sleep disorder is shown in the third regression model (path model c). Continuous variables including age, PA, sleep disorder and mental health were standardized. The mediated effect was examined with 95% bootstrapped confidence intervals (CIs), using 5,000 bootstrapped samples and bootstrap bias-corrected confidence intervals from the models were reported (Efron, 1987). The percentage of mediation was calculated by the equation = 100*indirect effect/total effect. Statistical significance was set at 0.05. All analyzes were performed using the R program (4.1 version, The R Project for Statistical Computing).^2^ The lavaan package in R was used to perform the mediation analyzes (Rosseel, 2011).

## Results

### Basic information of the participants

Of the final analytical sample (*n* = 2084), 1,274 (61.1%) were male and 810 (38.9%) were female (Table 1). By discipline, 1,363 students (65.4%) were majoring in Technology, 394 students (18.9%) were majoring in Natural Science, and 327 students (15.7%) were majoring in Liberal Arts, Medicine and Agricultural. By college degree, 985 students (47.3%) were undergraduate, 680 students (32.6%) were postgraduate, 419 (20.1%) were doctoral students. The age range of the participants was 18–35 years, and the mean BMI was 22.2 (95%CI, 22.1–22.4).

**Table 1:** Participants’ demographic characteristics.

	Total (*N* = 2084)
	Mean (95%CI)
Age	22.4 (22.2–22.5)
Height (cm)	171.5 (171.1–171.8)
Weight (kg)	65.8 (65.1–66.4)
Body Mass Index (BMI, kg/m^2^)	22.2 (22.1–22.4)
**Number (proportion)**	**No. (%)**
*Gender*	
Male	1,274 (61.1)
Female	810 (38.9)
*Discipline*	
Technology	1,363 (65.4)
Natural Science	394 (18.9)
Liberal Arts & Medicine & Agricultural	327 (15.7)
*College degree*	
Undergraduate	985 (47.3)
Postgraduate	680 (32.6)
Doctoral student	419 (20.1)
*Number of roommates*	
Zero	172 (8.3)
One	694 (33.3)
Two	628 (30.1)
Three	590 (28.3)

During the lockdown period, 172 (8.3%) students lived in a dormitory without roommate, 694 (33.3%) students shared a dormitory with one roommate (2 persons in a room), 628 students (30.1%) lived with two roommates (3 persons in a room), and 590 students (28.3%) had three roommates (4 persons in a room). The dormitories are similar in size, with an average size of 15 square meters each. As the proportion of students living without roommate was relatively small, therefore, we combined them with students who had one roommate in the subsequent analysis of the results.

### Changes in PA and SB

**Figure 2:**
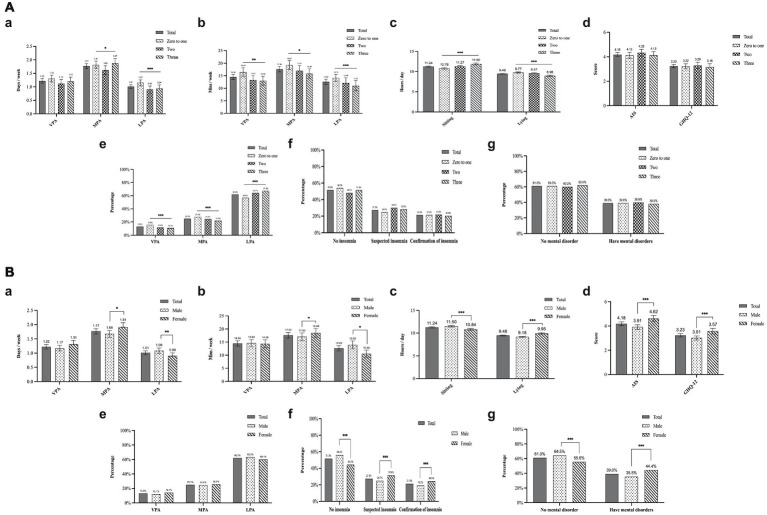
**(A)** PA behavior, sleep disorder, and mental health during the lockdown among different living conditions. ^*^p < 0.05, ^**^p < 0.01, ^***^p < 0.001. **(B)** PA behavior, sleep disorder, and mental health during the lockdown among gender. ^*^p < 0.05, ^**^p < 0.01, ^***^p < 0.001.

The average number of days that students were physically active at vigorous intensity during the lockdown period was 1.22 times, with a duration of 14.5 min (Figure 2A). The average number of times of moderate-intensity PA was 1.77 times, and the duration was 17.6 min, and the average number of times light-intensity PA was 1.01 times and the duration was 12.6 min. The average daily sedentary time was 11.2 h and lying time was 9.5 h (Figure 2A).

There was a significant gender difference in the duration of moderate and light-intensity PA, with female students having more moderate physical activity participation time than male students, and male students having more light-intensity physical activity engagement time than female students (Figure 2Bb).

In addition, male students had more sedentary hours than female students. Conversely, female students spent more time lying down than male students (Figure 2Bc). All PA profiles before and during lockdown are presented in detail in Table 2. There was a significant reduction in the total weekly PA level during lockdown (63.9%). The mean daily sedentary time increased by 21.4% and the mean daily lying time increased by 10.7% compared to the before lockdown period (*p* < 0.001). There were differences in the changes of different levels of intense physical activity between male and female students. Vigorous-intensity physical activity and moderate-intensity physical activity decreased by 52.4 and 63.2% for male students, respectively, while in females the corresponding changes were smaller at 36.9 and 46.9%, for vigorous intensity and moderate-intensity physical activity, respectively. In addition, the change in light-intensity physical activity was bigger among female students than male students (88.2% vs. 82.3%, *p* < 0.001).

**Table 2:** Changes in physical activity behavior.

		Total (*N* = 2084) Mean (95%CI)	Gender		Number of roommates		
			Male (*N* = 1,274) Mean (95%CI)	Female (*N* = 810) Mean (95%CI)	Zero to one (*N* = 866) Mean (95%CI)	Two (*N* = 628) Mean (95%CI)	Three (*N* = 590) Mean (95%CI)
Vigorous MET minutes/week	Pre	703 (653–753)	775 (712–838)	591 (508–674)	701 (620–781)	732 (632–832)	676 (596–755)
	Post	370 (333–407)	369 (318–419)	373 (321–425)	432.01 (368–496)	326.37 (266–387)	326.1 (263–389)
	∆%	47.3	52.4	36.9	38.3	55.4	51.8
	*p*–value	<0.001	<0.001	<0.001	<0.001	<0.001	<0.001
Moderate MET minutes/week	Pre	553 (522–584)	578 (538–619)	512 (463–561)	542 (495–590)	566 (505–626)	554 (499–608)
	Post	236 (216–256)	213 (190–236)	272 (236–308)	260 (227–293)	231 (191–272)	205 (179–232)
	∆%	57.3	63.2	46.9	52.1	59.1	62.9
	*p*–value	<0.001	<0.001	<0.001	<0.001	<0.001	<0.001
Light MET minutes/week	Pre	740 (709–770)	729 (691–768)	756 (706–805)	763 (712–813)	724 (670–779)	722 (670–774)
	Post	114 (100–127)	129 (110–148)	89.0 (72.4–106)	122 (103–141)	115 (87.7–143)	99.4 (74.5–124)
	∆%	84.6	82.3	88.2	84.0	84.1	86.2
	*p*–value	<0.001	<0.001	<0.001	<0.001	<0.001	<0.001
Sitting hours/day	Pre	9.26 (9.13–9.39)	9.37 (9.21–9.54)	9.08 (8.88–9.28)	9.06 (8.86–9.26)	9.32 (9.10–9.54)	9.49 (9.24–9.73)
	Post	11.2 (11.1–11.4)	11.5 (11.3–11.7)	10.8 (10.6–11.1)	10.8 (10.6–11.0)	11.3 (11.0–11.5)	11.9 (11.7–12.1)
	∆%	21.4	22.7	19.4	19.0	20.9	25.4
	*p*–value	<0.001	<0.001	<0.001	<0.001	<0.001	<0.001
Lying hours/day	Pre	8.56 (8.49–8.63)	8.48 (8.39–8.57)	8.69 (8.57–8.80)	8.63 (8.53–8.73)	8.75 (8.62–8.88)	8.25 (8.11–8.4)
	Post	9.48 (9.38–9.58)	9.18 (9.07–9.29)	9.95 (9.78–10.1)	9.77 (9.62–9.92)	9.57 (9.39–9.75)	8.96 (8.8–9.12)
	∆%	10.7	8.3	14.5	13.2	9.4	8.6
	*p*–value	<0.001	<0.001	<0.001	<0.001	<0.001	<0.001
Total PA	Pre	1995 (1907–2083)	2082 (1968–2,196)	1858 (1721–1996)	2006 (1865–2,146)	2022 (1853–2,190)	1952 (1804–3,000)
	Post	720 (662–777)	710 (632–788)	734 (649–819)	814 (715–912)	673 (567–779)	631 (540–721)
	∆%	63.9	65.9	60.5	59.4	66.7	67.7
	*p*–value	<0.001	<0.001	<0.001	<0.001	<0.001	<0.001

PA, physical activity.

### Changes in health condition

**Figure 3:**
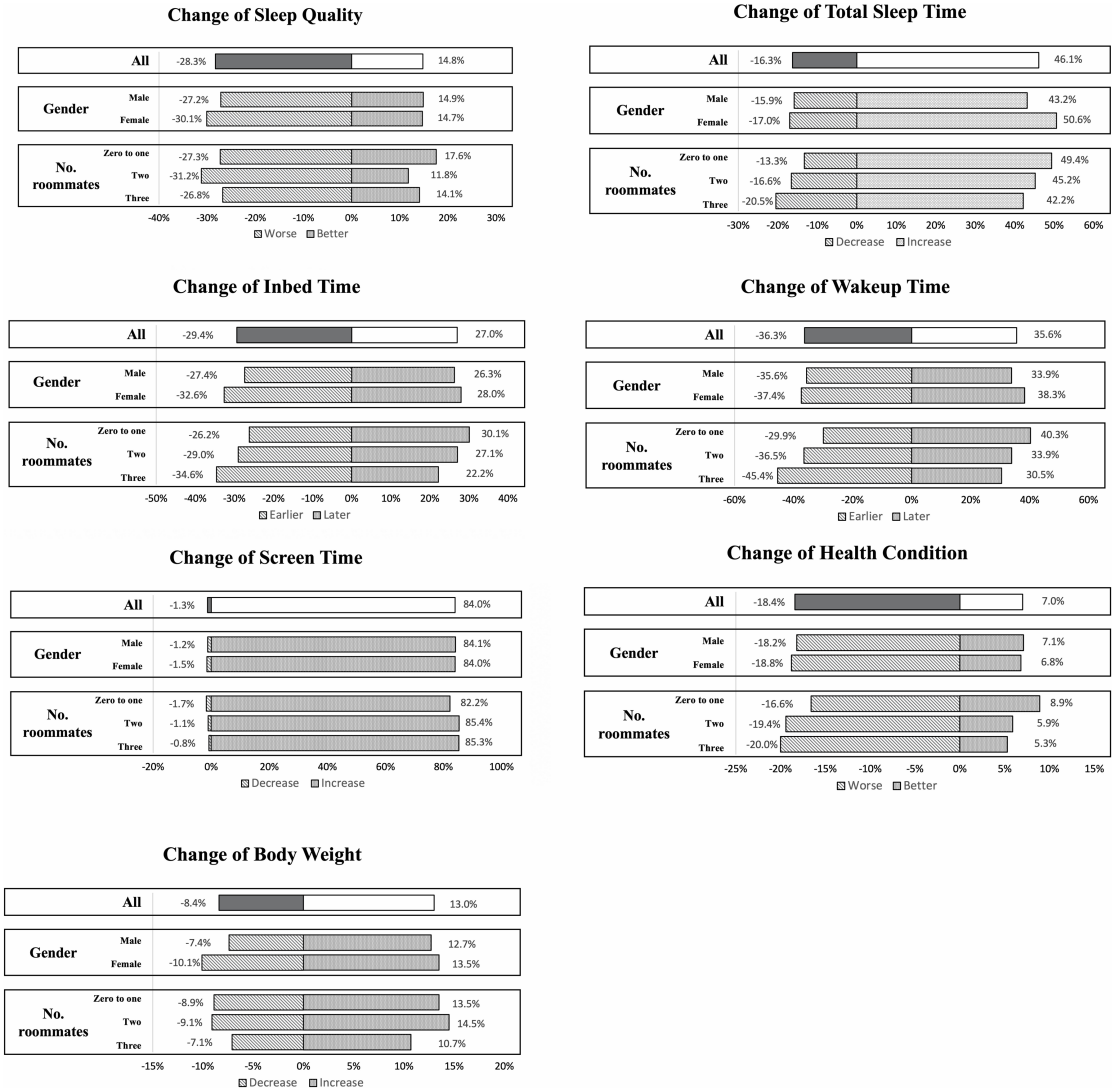
Changes in health behavior and condition among different gender and living environments.

Most of the students (74.6%) reported that their health condition was the same as before the lockdown, while nearly 18.4% reported a decrease in health condition and 7% reported better condition than before the lockdown. Nearly 59% of students reported no change in their weight compared to before the lockdown, 19.5% were unclear about their weight change, 13.0% reported weight gain, and 8.4% reported weight loss. On changes in screen time, nearly 84% of students reported an increase in their daily screen time from before the lockdown, about 14.7% reported no change in screen time, and nearly 1.3% reported a decrease in screen time from before the lockdown (Figure 3).

In addition, sleep behavior also changed significantly during the lockdown period. Compared to before the lockdown period, nearly 29.4% of students reported going to bed earlier and 27% of students reported going to bed later, while 43.6% reported no change in bedtime. Nearly 35.6% of students reported waking up later, 46.1% reported an increase in the total number of hours of sleep and 28.3% reported poorer sleep quality.

There were gender differences in sleep behavior changes during the lockdown. Female students had a higher percentage of earlier bedtimes and later wake-up bedtimes than male students, and a greater percentage of females reported an increase in total sleep time than male students during the lockdown period (*p* < 0.001). The mean score of the Athens Insomnia Scale was 4.18 (Figure 2Bd). Compared to male students, female students had significantly higher AIS scores, and experienced worse sleep disorders. In addition, nearly 31.4% of female students were diagnosed with suspected insomnia and nearly 24.3% were diagnosed with insomnia, while male students reported significantly less sleep disorder than female students (Figure 2Bf).

Regarding mental health during the lockdown period, female students scored significantly higher on average on the GHQ-12 scale than male students, and nearly 44.4% of female students were diagnosed with a mental disorder as opposed to 35.5% of male students (Figure 2Bg).

### Influences on PA and health behaviors in living dormitory conditions

Differences in physical activity participation also existed by dormitory conditions (Figures 2Aa,b). Students without or with one roommate spent significantly more time participating in all intensity of activities than those with two and three roommates. However, their lying hours were significantly longer than those with two and three roommates.

Those having one roommate or no roommate had less variation in total physical activity level than those having two roommates or having three roommates (59.4% vs. 66.7% vs. 67.7%). Also, similar differences between different dormitory conditions existed in changes of physical activity at different intensities.

Male students had more sedentary time compared to before the lockdown than female students. However female students had longer lying hours compared to before the lockdown than the male students. Students with three roommates had a greater change in sedentary hours than students in other dormitory conditions, spending more time sitting than before the lockdown. Those with one roommate or no roommate had a greater change in lying hours than those in other dormitory conditions, having more lying hours than before the lockdown. No differences were found in sleep and mental health by dormitory conditions (Figures 2Af,g).

Students with two and three roommates had worse health conditions during the lockdown compared to those with one and no roommates (19.4% vs. 20.0% vs. 16.6%, *p* = 0.029). In terms of change in bedtime, a greater proportion of students with three roommates reported going to bed earlier during the lockdown than those with one roommate or no roommate (34.6% vs. 26.2%, *p* = 0.002). In contrast, a larger proportion of students with one roommate and no roommate reported waking up later during lockdown than those with three roommates (40.3% vs. 30.5%, *p* < 0.001). On the change in total sleep time, students with three roommates reported a greater percentage of decreased total sleep time than students with two roommates and those with one roommate or no roommate (20.5% vs. 16.6% vs. 13.3%, *p* = 0.004). In turn, the proportion of students who had one roommate or no roommate and who had better sleep quality than before the lockdown was higher than those who had two roommates and those who had three roommates (17.6% vs. 11.8% vs. 14.1%, *p* = 0.016).

### Mediation effects of sleep disorder in the relationship between PA and mental health

The results of bootstrapped mediation models in different dormitory conditions after controlling for age and gender are presented in Table 3. In the path a, PA was negatively associated with sleep disorder in students who have three roommates. The total (path c) and direct (path c’) effects of PA on mental health in the model were significant when the dormitory conditions were having one roommate or no roommate, and having two roommates. In the group of students with three roommates, sleep disorder was positively associated with mental health (path b), although there was no significant association between PA and mental health (path c’).

**Table 3:** Mediation analyzes: association between physical activity and mental health *via* sleep disorder.

		Total effect model (mental health)					Mediator (sleep disorder)					Direct effect model (mental health)				
					Bootstrap 95% C.I.					Bootstrap 95% C.I.					Bootstrap 95% C.I.	
Group	Variables	*β*	SE	*p*-value	CI.L	CI.U	*β*	SE	*p*-value	CI.L	CI.U	*β*	SE	*p*-value	CI.L	CI.U
All	PA	−0.106	0.07	<0.001^***^	−0.481	−0.205	−0.072	0.079	0.001^***^	−0.411	−0.103	−0.064	0.056	<0.001^***^	−0.315	−0.095
	Sleep disorder											0.585	0.016	<0.001^***^	0.494	0.556
	Gender	0.083	0.146	<0.001^***^	0.261	0.834	0.097	0.163	<0.001	1.035	0.097	0.026	0.121	0.154	−0.075	0.401
	Age	0.033	0.024	0.137	−0.011	0.082	0.01	0.027	0.639	−0.04	0.065	0.027	0.019	0.123	−0.007	0.067
Zero to one	PA	−0.088	0.104	0.008^**^	−0.482	−0.074	−0.044	0.116	0.171	−0.387	0.075	−0.063	0.082	0.015^*^	−0.366	−0.043
	Sleep disorder											0.564	0.026	<0.001^***^	0.448	0.55
	Gender	0.083	0.222	0.013^*^	0.114	0.985	0.045	0.131	0.024^*^	0.041	0.551	0.039	0.184	0.166	−0.097	0.621
	Age	0.014	0.111	0.691	−0.173	0.261	0.001	0.067	0.991	−0.127	0.138	0.014	0.082	0.599	−0.112	0.208
Two	PA	−0.149	0.132	< 0.001^***^	−0.751	−0.233	−0.07	0.146	0.084	−0.529	0.051	−0.107	0.108	0.001^**^	−0.566	−0.139
	Sleep disorder											0.605	0.035	<0.001^***^	0.487	0.623
	Gender	0.064	0.259	0.104	−0.086	0.928	0.078	0.16	0.001^**^	0.234	0.853	−0.016	0.22	0.633	−0.525	0.321
	Age	0.063	0.17	0.232	−0.13	0.536	0.012	0.102	0.704	−0.156	0.245	0.05	0.141	0.242	−0.103	0.449
Three	PA	−0.090	0.138	0.035^*^	−0.563	−0.021	−0.114	0.145	0.005^**^	−0.686	−0.12	−0.022	0.114	0.525	−0.296	0.15
	Sleep disorder											0.593	0.03	<0.001^***^	0.476	0.595
	Gender	0.105	0.301	0.021^*^	0.107	1.29	0.048	0.175	0.066	−0.027	0.656	0.056	0.239	0.116	−0.072	0.855
	Age	0.235	0.283	0.007^**^	0.204	1.31	0.119	0.152	0.011^*^	0.093	0.694	0.114	0.24	0.122	−0.106	0.834

PA, physical activity level during the lockdown; Mental health, General Health Questionnaire-12 items; Sleep disorder, Athens Insomnia Scale. β: Path coefficients; SE: Standard Error; CI.L: Lower limit of 95% Confidence interval; CI.U: Upper limit of 95% Confidence interval.

* *p* < 0.05;

** *p* < 0.01;

*** *p* < 0.001.

As shown in Table 4, sleep disorder did not mediate the relationship between PA and mental health in students who had one roommate or no roommate, whereas among students with two roommates there was a slight tendency for an indirect effect (*p* = 0.088), with a mediation rate of 28.2% (partial mediating effect). Only the bootstrapped CI of indirect effects in students who had three roommates was statistically significant, with 75.6% as a percentage of mediation (complete mediating effect). Specifically, sleep disorder mediated the relationships between PA and mental health.

**Table 4:** Total, direct, and indirect effects of the mediation analyzes investigating sleep disorder as a mediator between physical activity and mental health.

					Bootstrap 95% C.I.		
Group	Model	*β*	SE	*p*-value	CI.L	CI.U	Percent of mediation (%)
All	Total effect	−0.106	0.07	<0.001^***^	−0.481	−0.205	
	Indirect effect	−0.042	0.04	<0.001^***^	−0.214	−0.056	39.6
	Direct effect	−0.064	0.056	<0.001^***^	−0.315	−0.095	60.4
Zero to one	Total effect	−0.088	0.104	0.008^**^	−0.482	−0.074	-
	Indirect effect	−0.025	0.058	0.172	−0.193	0.038	-
	Direct effect	−0.063	0.082	0.015^*^	−0.366	−0.043	-
Two	Total effect	−0.149	0.132	<0.001^***^	−0.751	−0.233	-
	Indirect effect	−0.042	0.082	0.088	−0.295	0.028	28.2
	Direct effect	−0.107	0.108	<0.001^***^	−0.378	−0.066	71.8
Three	Total effect	−0.090	0.138	0.035^*^	−0.563	−0.021	-
	Indirect effect	−0.068	0.08	0.006^**^	−0.378	−0.066	75.6
	Direct effect	−0.022	0.114	0.525	−0.296	0.15	24.4

β: Path coefficients; SE: Standard error; CI.L: Lower limit of 95% confidence interval; CI.U: Upper limit of 95% confidence interval; Percent of mediation = 100 * indirect effect/total effect.

* *P* < 0.05;

** *P* < 0.01;

*** *P* < 0.001.

## Discussion

In this study, more than two-thousand university students responded to our online survey on their PA, SB, and health behaviors. The results indicated significant reductions in participation time for all three intensity levels of PA compared to before the lockdown, and are largely consistent with those previously observed in university students (Wathelet et al., 2020; DeYoung and Li, 2022; Nirala et al., 2022). Dormitory living condition (number of roommates) was found to be a key factor affecting the students’ PA level during the isolation period, and also regulated the mediating role of sleep disorders in PA and mental health. These results suggest that the PA of university students isolated in dormitories is substantially affected by their living conditions.

### The PA and SB pattern during the strict lockdown period

Students’ total weekly energy expenditure in PA decreased by 63.9%, and sedentary and lying time increased by 21.4 and 10.7%, respectively during lockdown. These results are in agreement with those observed in earlier studies (Wang et al., 2020; Li et al., 2021; Alshammari et al., 2022). A study investigating the PA and SB of the young adult in Kosovo during COVID-19 confinement found that the total weekly energy expenditure of respondents decreased by 26.2% compared with that before the confinement. Of note is that the sample of this Kosovo study were isolated at home, and had more opportunities for housework and other activities than the university students who were in dormitory lockdown in our study. These university students had all their meals delivered to their door by volunteers, and they did not participate in household chores.

### Gender differences in sleep and mental health during the lockdown period

The current study found that female students showed greater variation in sleep habits than male students during the COVID-19 lockdown. Their sleep and mental health were also poorer than those of male students. These results mirrored the findings of previous studies that female students experienced greater insomnia (Zhou et al., 2020; Tasso et al., 2021), anxiety and depressive symptoms (Pieh et al., 2020; Al Mamun et al., 2021; Lewis et al., 2021). A global study examining sleep patterns based on wearable devices found that even though women have longer sleep duration, they have more nighttime awakenings at any age and are also at higher risk of sleep disorders and insomnia than men (Jonasdottir et al., 2020; Otten et al., 2021). A possible explanation is that women may be more prone to stress and anxiety when faced with some traumatic events (McLean et al., 2011; Al Mamun et al., 2021) and that this affects their sleep behavior, while men show better resilience and consistency (Axinn et al., 2013). The evidence suggests that there are consistent gender differences in both sleep and mental health, and further research should focus more on effective interventions to improve women’s sleep and mental health.

### Effects of living conditions on PA, and the mediation role of sleep among PA and mental health

Previous studies have found that university students had significantly lower physical activity during the pandemic lockdown and worse sleep and psychological state than in normal life (Wang et al., 2020; Hargreaves et al., 2021; Lewis et al., 2021; Wilson et al., 2021). Our study is the first to use university students’ living conditions context as a factor to explore changes in PA, sleep and mental health during a COVID-19 lockdown. Importantly, the PA differences we observed across living conditions only existed during lockdown period. We found that physical activity decreased with number of roommates, with those with more roommates reporting less physical activity time (Hargreaves et al., 2021). This differs from most previous research settings that have used Chinese university students as participants (Deng et al., 2020). The environmental specificity of Chinese university dormitories, which have a high density of indoor occupancy (Evans et al., 2001), with students sharing with other students (Tao et al., 2016) and having very limited space to move around aside from beds and desks and closets, tends to increases sedentary and lying hours among students under lockdown. In addition, confronted with lockdown restrictions in dormitories with limited space, the motivation to engage in PA is likely to decline. Similarly, PA changes were more significant for those living in apartments than those living in other areas and PA was significantly lower than before the lockdown (Aguilar-Farias et al., 2020). Our findings show that students who live in a dormitory alone or share with one other student have higher PA levels than students who share a dorm with 2 or 3 other students. The dorm sizes are similar, thus more roommates results in less living space for each student. Therefore, the decrease in PA was mainly attributed to the limited physical space and the students not being used to exercising in dormitories. This finding urges us to develop novel approaches to promote PA among university students in such limited living conditions in the post-pandemic era.

Little is known about the relationship between PA, sleep, and mental health among Chinese university students in COVID-19 dormitory lockdown. To clarify the role of PA on the relationship between sleep disorder and mental health, we further conducted a series of mediation analyzes and found that PA had a positive impact on sleep and mental health. In normal life without the COVID-19 pandemic, the severity of insomnia symptoms has been shown to be associated with an increase in mental health symptoms such as depression and anxiety (Alvaro et al., 2013; Lewis et al., 2021). Increasing PA can improve the sleep health of university students and also regulate their mental health (Wu et al., 2015). Notably, our findings align with those of previous studies (Teychenne et al., 2008) that PA was a predictor of mental distress and PA was negatively associated with depression symptoms caused by COVID-19 lockdown (Deng et al., 2020; Brailovskaia et al., 2021).

Most notably, sleep disorder became relevant in predicting the indirect relationship between PA and mental health during the COVID-19 lockdown period. This finding is consistent with that of Lewis et al., who found that individuals with high levels of PA reported fewer symptoms of insomnia, which in turn was associated with fewer symptoms of depression and anxiety (Lewis et al., 2021). However, they also found that this indirect relationship did not exist before lockdown. This result may be explained by the negative psychological effects of lockdown, and the absence of various outlets for stress (Brooks et al., 2020). Furthermore, it is worth noting that this indirect relationship is only present in students with more than two and three roommates. This is consistent with our hypothesis and is likely due to the overcrowding of available space per student, highlighting the positive effects of PA on mental health. Even though the size of the indirect effect was moderate, despite worse sleep quality, students may experience a further improvement in mental wellness if they are more physically-active. Therefore, students who are isolated in university dormitories such as indoor high-density living environments during COVID-19 lockdown should keep physically active and keep good sleep habits to reduce the risk of insomnia and mental disorders.

### Limitations and strengths of the study

The main limitation of the present study was not able to determine causal relationships from the cross-sectional design. Second, the participants need to recall their daily activity and sleep habits before the lockdown, and this study could not observe the actual volume of daily activity and sleep habits using objective markers. Third, our participants were from the same university which may be not representative of the entire Chinese university student population. Besides, reported subjective information such as height and weight may be prone to recall bias. Further study may use wearable devices to obtain accurate behavioral and mental health data when conditions permit. Despite above mentioned limitations, this study has several strengths. To the best of our knowledge, this is the first study to investigate the PA, sleep and mental health of Chinese university students after nearly 1-month of dormitory isolation during COVID-19. The present study extends the previous results on PA, sleep, and psychological conditions and better explains the impact of strict quarantine on dormitory-resident university students. The findings may also provide a timely and helpful report on the challenge of lifestyle change and psychological disturbances to relevant university and student bodies. Moreover, this study explored the number of roommates that might evoke various experiences and conditions during the lockdown.

## Conclusion

This study showed that strict lockdown during the COVID-19 pandemic caused a significant decrease in physical activity and altered daily routines such as sleep, which had a negative impact on the health behavior among university students living in dormitories. Even with limited space, engaging in PA was associated with better sleep and mental health. Sleep disorder mediated the relationship between PA and mental health when the number of students in the dormitory was three and four. These results may provide useful suggestions for relevant authorities, for example to adjust the student dormitory occupancy to one or two students sharing a room, and to take steps to encourage students to exercise in a limited space.

### Practical implications

The reported reduction in participation time for all levels of PA might affect students’ well-being. Therefore, the universities should continue cooperation among units to embed health and physical literacy in fostering students’ knowledge and skills to maintain an active lifestyle during pandemics or other public health emergencies. The findings indicated that the living environment might affect students’ health during confinement, and nonetheless suggest universities and stakeholders relocate residents and adopt to single or twin rooms in the early stage of lockdown and suggest offering students more space with adequate privacy where is possible. Moreover, providing timely access to psychological counseling and advice for female students may minimize the adverse effects on their health and wellness. Furthermore, identify the early sign of distress and give appropriate services to safeguard university students’ holistic health during a public health crisis.

## Data availability statement

The original contributions presented in the study are included in the article/supplementary material, further inquiries can be directed to the corresponding author.

## Ethics statement

The studies involving human participants were reviewed and approved by Shanghai Jiao Tong University. Written informed consent was obtained from all participants for their participation in this study.

## Author contributions

SC, XW, and BZ developed the idea of the manuscript. BZ, SML, SC, and XW drafted the paper plan and conducted manuscript preparation, review, and revision. QG and SL participated in data collection and reviewed the manuscript. All authors contributed to the article and approved the submitted version.

## Funding

The study was funded by the start-up plan for new young teachers grant (Grant AF4150043) from Shanghai Jiao Tong University, China.

## Conflict of interest

The authors declare that the research was conducted in the absence of any commercial or financial relationships that could be construed as a potential conflict of interest.

## Publisher’s note

All claims expressed in this article are solely those of the authors and do not necessarily represent those of their affiliated organizations, or those of the publisher, the editors and the reviewers. Any product that may be evaluated in this article, or claim that may be made by its manufacturer, is not guaranteed or endorsed by the publisher.

## Footnotes

^1^ https://www.wenjuan.com

^2^ https://www.R-project.org/
